# International experience of a direct supervisor–does it matter for self-initiated expatriates’ adjustment?

**DOI:** 10.1371/journal.pone.0326848

**Published:** 2025-06-23

**Authors:** Irma Baneviciene, Luisa Helena Pinto, Vilmante Kumpikaite-Valiuniene

**Affiliations:** 1 School of Economics and Business, Kaunas University of Technology, Kaunas, Lithuania; 2 School of Economics, University of Porto, Porto, Portugal; IUB: The Islamia University of Bahawalpur Pakistan, PAKISTAN

## Abstract

Due to increasing global mobility flows, self-initiated expatriates (SIEs) in both employee and managerial roles are now commonplace. However, the influence of direct supervisors’ international experience on the adjustment of SIEs remains underexplored. This study, grounded in signaling and similarity-attraction theories, addresses this gap through a qualitative examination of supervisors with international experience and at least one SIE under their supervision. The findings indicate that both foreign-born and locally born supervisors perceive their international experience as valuable in managing their international teams. Foreign-born supervisors, more frequently than their locally born counterparts, interpreted the uncertainty signals from their SIE employees as a reflection of empathy and open-mindedness, attributes shaped by their international backgrounds and cultural insights. Furthermore, the results suggest that all direct supervisors focus their support and actions primarily on facilitating SIEs’ adjustment in the work environment, rather than in the non-work environment. This research offers theoretical and practical insights for international human resource management, highlighting the positive impact of supervisors with international experience on the onboarding and adjustment processes of new SIEs, thereby enhancing the management of international teams.

## Introduction

This study addresses the growing global mobility trend and its implications for local organizations employing foreign-born employees. It focuses on self-initiated expatriates (SIEs), defined by Cerdin and Selmer [1] as individuals who voluntarily relocate to a foreign country for work without support from an employer. This research explores how the international experience of direct supervisors—whether foreign-born or locally born—affects the adjustment of SIEs working under their supervision.

Self-initiated expatriates (SIEs) encounter many of the same challenges as newly hired employees; however, these are compounded by the need to adapt simultaneously to unfamiliar cultural, organisational, and interpersonal contexts. A key factor influencing their adjustment is the social support received in the host environment, particularly from coworkers and direct supervisors [2–4]. Among the various organisational influences on SIE adjustment, the role of the direct supervisor emerges as especially significant. Recent studies suggest that supervisors with prior international experience are better positioned to recognise and respond to the specific needs of expatriates, offering tailored support that facilitates both professional integration and psychological well-being [5]. Consequently, supervisor support is often more instrumental in fostering work adjustment than peer support, as supervisors are typically more accessible for addressing task-related queries and providing performance guidance [6,7]. Similarly, mentors with international experience have also been shown to influence expatriate outcomes positively [8].

The importance of supervisory support becomes even more pronounced when considering regional variations in expatriate experiences. For example, European SIEs in China and Turkey report differing patterns of social interaction and psychological well-being, shaped by the varying degrees of cultural distance and regional familiarity [9]. In several Asian contexts—such as China, Taiwan, and South Korea—research indicates that SIEs benefit considerably when supervisors deeply understand the host country’s cultural and institutional dynamics, often informed by their own international exposure [10,11]. In sub-Saharan Africa, the presence of robust local support systems is essential for expatriate success [12,13]. While empirical evidence from Africa and Latin America remains more limited, existing studies underscore the universal relevance of culturally informed supervision. In these contexts as well, supervisors with global insight can play a pivotal role in bridging cultural divides and guiding SIEs through unfamiliar social and professional landscapes [14].

While these findings highlight the value of supervisor support, particularly when informed by international experience, there remains limited understanding of how such experience specifically shapes SIEs’ adjustment processes. Although some studies suggest that supervisors with global exposure are better equipped to provide effective support [6,7,15–18], the underlying mechanisms through which this occurs remain underexplored. To address this gap, the present study investigates **how the international experience of both local and foreign-born supervisors intersects with SIEs’ adjustment across work and non-work domains.**

The novelty of this study lies in its investigation of how direct supervisors interpret and act on the behaviours and uncertainty signals of SIEs. The research draws on signaling [19] and similarity-attraction theories [20] to propose that supervisors with international experience, whether foreign-born or locally born, are more attuned to the uncertainty signals of SIEs. Uncertainty signals encompass a range of verbal and non-verbal cues through which individuals convey doubt or a lack of clarity. These may include direct verbal expressions, such as questions aimed at seeking clarification, as well as non-linguistic features like hesitation, rising intonation, or speech disfluencies [21,22]. Non-verbal manifestations—such as gestures, body posture, and facial expressions—also play a crucial role in signalling uncertainty during interpersonal interactions [23,24]. These supervisors, recognising shared experiences, are more likely to offer support, thus facilitating SIEs’ adjustment. This is particularly pertinent as global mobility flows increase, resulting in a growing number of SIEs in local workforces, which, in turn, impacts management practices.

The contributions of this study are threefold. First, the theoretical implications align with signalling theory, suggesting that while SIEs may express uncertainty, these signals are not always directed at and noted by their supervisors. Supervisors with international experience are more likely to recognise and interpret these signals regardless of their national origin. However, foreign-born supervisors are more adept at identifying and interpreting SIE uncertainty signals than locally born supervisors, though the latter tend to take more actions to assist SIEs’ general adjustment. Further research is needed to explore why foreign-born supervisors, despite similar experiences, do not provide more comprehensive support.

Second, the managerial contributions of this study highlight the role of foreign-born supervisors in recognising SIE uncertainty signals and taking proactive steps to assist. A failure to recognise these signals could lead to misunderstandings, disrupting workplace dynamics. This finding underscores the importance of supervisor cultural diversity in organisations with incoming SIEs. While foreign-born supervisors may not always be familiar with working in multinational teams, they are more attuned to the adjustment challenges SIEs face, often initiating conversations or seeking HR assistance when needed.

Third, this study provides valuable insights for employers regarding managerial staffing strategies. The results demonstrate that foreign-born supervisors, with their initial connections to incoming SIEs, are more likely to reduce turnover and enhance productivity during the adjustment period. Therefore, employers should consider hiring SIEs into managerial positions to support the integration of new SIEs into the workforce. Moreover, from a practical standpoint, this research suggests that Human Resource Management (HRM) professionals need to adopt more strategic, interventionist roles, employing tools such as cross-cultural support, regular communication, and culturally diverse mentoring to help ease SIEs’ transitions. Encouraging supervisors to leverage their international experience to support SIEs can facilitate smoother adjustments and increase workplace performance.

The article proceeds as follows: a theoretical framework outlining signalling and similarity-attraction theories; a review of the literature on expatriates’ international experience and cross-cultural adjustment, with a focus on the supervisor-SIE relationship; a description of the methodology, including research design, data collection procedures, participant information, and data analysis; and finally, a discussion of the findings, limitations, future research directions, and practical implications.

## Theoretical framework

### Signaling theory

Signaling theory was first formulated by Spence [19] based on the signaling function of job applicants’ education to potential employers. Since then, it has often been used to research various phenomena in business and management. The primary elements in signaling theory are the *signaler*, the *receiver*, and the *signal* itself [25]. When scholars discuss the signaler in their studies, they differentiate between credible, reliable, and inferior “cheat” signals the signaler sends [26–28]. Signaling theory scholars also emphasize the receiver’s attention to the signals and their interpretation [29–31]. The studies in the management field primarily focus on signalers and receivers who are interested in the processes within the organization, including recruiters [32–34], managers [35–37], employees [38–40], and corporate headquarters and subsidiaries [41,42].

In our study, the newcomer *SIE is a signaler*. As SIEs enter a new foreign workplace, they presumably send uncertainty signals. Their uncertainty arises because they are new to the organizational culture, its requirements, and the relationships with coworkers. Therefore, they ask questions and seek assistance from their direct supervisor or coworkers. Also, incoming SIEs observe and try to gauge others’ reactions to their behavior, appearance, or use of language. Doing so makes them most likely to send uncertainty signals by appearing too cautious or overly excited. Although the signals are not necessarily targeted at the direct supervisor, *a direct supervisor with international experience as a signal receiver* will likely notice and interpret them based on their personality and experience. Through the similarity-attraction perspective, we view a direct supervisor’s international experience as driving the supervisor to pay more attention to SIE uncertainty signals and expand their scope of interpretation.

### Similarity-attraction theory

Similarity-attraction theory posits that people tend to have more positive interactions with others who share similar characteristics [20,43,44]. The relationship dynamics between direct supervisors with international experience and SIEs can be influenced by their shared international experiences [20,45]. This international background provides supervisors with an additional lens through which they perceive SIEs upon joining the organization. Despite potential differences in the nature and extent of their international experiences, this shared background becomes an initial and easily recognizable point of commonality between the SIE and their direct supervisor during the early stages of their working relationship. This, in turn, should be helpful for SIEs’ adjustment.

### International experience and cross-cultural adjustment

International experience encompasses the various experiences gained through working, living, studying, and traveling abroad [46]. Takeuchi et al. [47] examined the impact of international experiences on assigned expatriates (AEs) and their cross-cultural adjustment. They differentiated between previous work experiences (in terms of number and length) and travel experiences. They discovered that prior international experiences do not directly cause cross-cultural adjustment but moderate it [47]. Dimitrova et al. [48] adopted a Job Demands-Resources perspective, suggesting that international experience contributes to resources like social and human capital, which can facilitate adjustment to new assignments. Grill et al. [49] highlighted that international cross-cultural experience primarily impacts the adjustment of inexperienced expatriates. However, Fenner and Selmer [50] found no significant relationship between international experience and expatriates’ psychological adjustment in a study investigating expatriates’ adjustment across public and private sectors.

These controversies in the literature underscore the relevance of studying the effects of international experience on SIEs. In this context, the international experience of both direct supervisors and employees can serve as a mutual understanding of the difficulties associated with living and working abroad. The argument is that the international experience of direct supervisors and SIEs can bring them closer together, enhancing SIEs’ adjustment in both work and non-work environments.

### Supervisor-SIE relationship

Direct supervisors play a critical role in communication and the implementation of organizational strategy [51–53], and the supervisor-employee relationship has been a longstanding focus of research. This relationship is multifaceted [54], with extensive research examining how newcomers interact with their managers. Key areas explored include socialization behaviours [55–57], leadership styles [58,59], trust and support [60–62], expectations and experiences of both newcomers and supervisors [59,63], and creativity among newcomers [64].

Self-initiated expatriates (SIEs), who enter the foreign workforce, are also newcomers to an organisation, whether domestic or multinational. These individuals bring diverse cultural backgrounds, socialisation practices, languages, and expectations, which complicate communication within multicultural teams [65]. The dynamics between supervisors and employees in these teams introduce additional challenges, likely influencing SIEs’ adjustment. Existing research on supervisor-employee relationships, particularly in management and global mobility contexts, largely focuses on assigned expatriates in managerial roles [66–68] and their local subordinates [69–72].

The role of a direct supervisor’s international experience in SIEs’ adjustment holds significant importance for several reasons. According to Kaur et al. [15], SIEs perceive supervisors with international experience as more reliable in fostering trust and establishing supportive interpersonal relationships, which are crucial for adjustment. Supervisors with international experience, equipped with knowledge of historical, legal, and employment contexts, are better positioned to facilitate smoother transitions and align expectations for SIEs within an organization [17]. From a human resource management perspective, hiring supervisors with international experience ensures readiness to support incoming SIEs and reduce adjustment-related challenges [16,18].

Takeuchi and Chen [46] have called for further research across different contexts to explore how the international experience of direct supervisors influences the adjustment of SIEs. This study responds to this call by investigating the impact of direct supervisors’ international experience on SIEs’ adjustment in both work and non-work environments, drawing on signaling [19,25] and similarity-attraction [43] theories. It is suggested that direct supervisors with international experience develop a unique relationship with subordinate SIEs, characterised by supportive attitudes and higher levels of social support, which positively influences SIEs’ adjustment in both work and non-work environments.

## Methodology

### Research design

The study follows a qualitative research methodology [73], which uncovers deeper processes in individuals, teams, and organizations and allows researchers to understand what individuals experience and how they interpret their experiences [74]. Given the study’s focus on understanding the subjective experiences and socially constructed meanings of SIEs, this research adopts an interpretivist philosophical stance [75,76]. Interpretivism is particularly well-suited for qualitative inquiry as it emphasizes contextual understanding, researcher reflexivity, and the co-construction of meaning between researchers and participants [77]. This approach allows for the exploration of complex social phenomena, such as cross-cultural adjustment, within their natural settings, where individual perceptions, meanings, and interactions are central. It supports a flexible, nuanced examination of how SIEs and their supervisors navigate cultural and institutional environments.

### Procedures of data collection

For this study, 20 semi-structured interviews were conducted with direct supervisors with international experience who also met some other selection criteria: (i) being an SIE in the US for more than ten years or (ii) being locally-born but having international experience, such as engaging in extensive international activities like studying, working, or traveling abroad for extensive periods. Furthermore, all participants should have at least one SIE (i.e., a foreign worker legally employed and displaced in the US on their own initiative) under their supervision. The United States was chosen for this study as it is a multicultural country, and local organizations are more likely to hire SIEs. In this study, the United States is considered the “home country” to analyze the experiences of foreign-born or locally born supervisors with international experience outside the US, and the “host country” for the SIEs under their supervision. Following initial contact with local supervisors who had international experience through the first author’s network, a snowball sampling technique was used to identify additional interviewees who met the selection criteria. Data collection was stopped when thematic saturation was achieved [73]. Purposeful sampling, with a specific focus on managers supervising self-initiated expatriates within a particular regional context, facilitated the achievement of both thematic and meaning saturation. Thematic saturation was reached when no new codes or themes emerged from the data, while meaning saturation—defined as obtaining a rich, nuanced understanding of the identified themes—was also attained. These procedures are consistent with previous research, which suggests that 16–24 interviews are typically sufficient to reach both types of saturation in qualitative studies [78]. The sample size of 20 is considered adequate to comprehensively address the research question in qualitative research with narrowly defined research objects [79–81].

The research has been approved in accordance with Kaunas University of Technology’s Description of the Procedure for Ethical Assessment of Research, as approved by the Kaunas University of Technology Rector’s Order No. A-201 of 23 April 2021, and the Kaunas University of Technology Research Ethics Commission Protocol No. M4-2022-15 of 28 October 2022. The interviews, conducted from November 1, 2022, to May 31, 2023, aimed to capture the insights and perspectives of direct supervisors who could provide firsthand knowledge of managing SIEs within the US context (see the interview protocol in S1 Appendix). All interviews were conducted in English and were done by the same researcher. Interviews were conducted face-to-face or via video conference, recorded, and transcribed for analysis. Before each interview, verbal permission to record was obtained, and this permission was recorded along with the interview. Each interviewee was free to refuse to answer any question if they felt uncomfortable sharing information and to withdraw from the study at any time, during or after the interview. Also, interviewees were assured of the confidentiality and privacy of collected data. The average duration of the interview was 27 minutes.

### Research participants

In this study, a cohort of 20 respondents actively participated. The age ranged from 30 to 63 years old, with ten individuals of each gender. Six respondents were locally born Americans with diverse international experiences, while fourteen were foreign-born. Furthermore, all participants had accumulated significant supervisory experience, ranging from 2 to 14 years. They all managed teams that included at least one SIE who was not of the same nationality. All foreign-born participants held undergraduate or graduate degrees in higher education, whereas half of the US-born supervisors had high school diplomas. These demographics provided a diverse and well-rounded participant pool for gathering insights on how direct supervisors with international experience manage SIEs. Information and details of the interviewees are summarised in S2 Appendix.

### Inclusivity in global research

Additional information regarding the ethical, cultural, and scientific considerations specific to inclusivity in global research is included in the Supporting Information (SX Checklist).

### Data analysis

**Fig 1:**
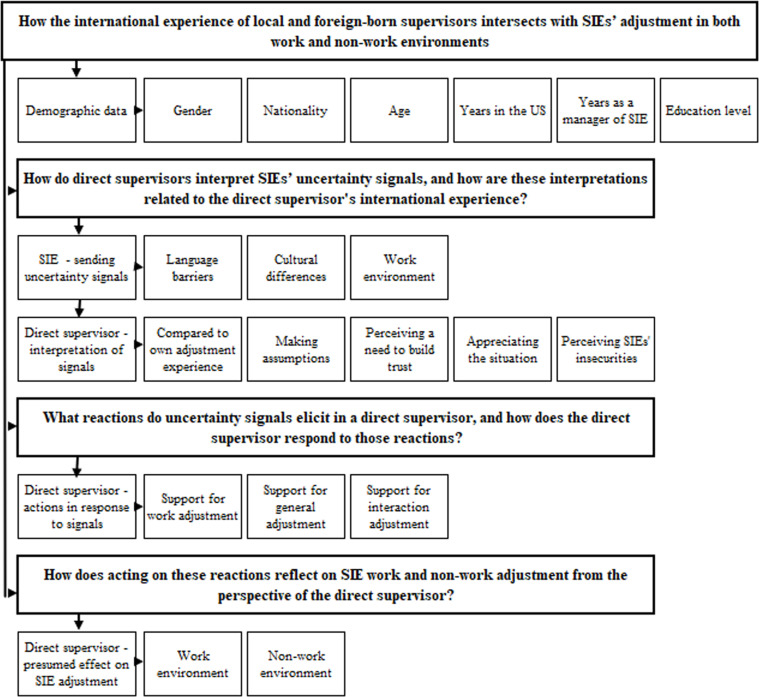
Thematic coding structure.

MAXQDA software was used to analyze and code the transcribed interviews to ensure transparency and reliability in the coding process. A thematic content analysis was conducted, employing both inductive and deductive coding approaches [82]. A thematic coding structure was developed in multiple steps to ensure systematic analysis (presented in Fig 1). First, demographic data were coded, including gender, nationality, age group, years in the United States, years as a supervisor of a multinational team, and educational level. Second, the main thematic coding categories were developed based on the study’s research questions. Third, these main themes were further subdivided into sub-themes through an inductive, data-driven approach informed by data interpretation. To support coding transparency and consistency, collaborative practices were followed as suggested by Ortloff et al. [83]. All coding decisions were discussed among all co-authors, and any discrepancies in interpretation were resolved through consensus. This collaborative process also ensured analyst triangulation, enhancing the trustworthiness of the findings. The lead author’s extensive experience working with and supervising SIEs within the U.S. context supported the initial data interpretation.

Following the development of main themes, all authors reviewed and refined the coding, resulting in an agreed-upon final code list [84]. This collaborative approach strengthened the reliability and validity of the qualitative analysis. The grouping was based on the facets of the Black et al. expatriate adjustment model, which includes work adjustment, general adjustment, and interaction adjustment [85]. Following, an additional categorisation was introduced, distinguishing between adjustments in work and non-work environments [85,86].

In line with the interpretivist paradigm underpinning this study, the positionality of the researchers and the reflexive nature of qualitative inquiry are acknowledged. Given varying degrees of intercultural experience and familiarity with expatriate contexts, the researchers were positioned not as detached observers but as active participants in the co-construction of knowledge. Interpretations were shaped by cultural backgrounds, disciplinary training, and pre-existing assumptions concerning global mobility and adjustment. To address this, continuous reflexivity was embedded throughout the research process, particularly during data collection and analysis, to examine critically how researcher perspectives may have influenced participant interactions and the interpretation of narratives. The use of reflective field notes and peer debriefings contributed to the mitigation of potential biases and supported the credibility and transparency of the study’s findings.

## Findings

The following key findings distinguish between two main categories of supervisors, all of whom possess international experience: (i) those born abroad, and (ii) those of local origin who have gained such experience through studying, working, or residing for extended periods in foreign contexts. In each table, the numeric data refers to the total number of respondents and references, organized in descending order. Several subsidiary questions were developed to explore the main research question: How does the international experience of a direct supervisor influence the adjustment of self-initiated expatriates (SIEs) in work and non-work environments?

**How do direct supervisors interpret SIEs’ uncertainty signals, and how are these interpretations related to the direct supervisor’s international experience?**

Table 1 presents the main uncertainty signals that the foreign-born and locally born direct supervisors observed, which we grouped into three categories: language barriers, cultural differences, and work environment.

**Table 1:** The codes of the main uncertainty signals observed by the direct supervisor.

Uncertainty signals	Foreign-born supervisors		Locally-born supervisors	
	Individuals reporting the reference	Number of references made	Individuals reporting the reference	Number of references made
**Language barriers**				
Poor English language	8	12	5	7
Thick accent	3	6	0	0
**Cultural differences**				
Behavior	8	12	2	2
Appearance	2	4	0	0
Food	1	1	1	1
**Work environment**				
Work ethic	8	14	3	6
Organizational culture	4	7	0	0
Complaints of inequality	1	4	0	0
No basic understanding of work processes	1	1	0	0

One of the most frequently observed uncertainty signals noted by all supervisors was *poor English language skills*, identified by 13 participants. The US is an English-speaking country, and English is the official business language; therefore, newcomers are expected to have sufficient English proficiency to communicate effectively. Therefore, most incoming SIEs are learning English as a second language, which may lead to initial communication challenges. The results confirm that:

*“English is not their first language, so conveying information to them can be difficult. It seems they understand English well, but trying to get it out and that <> colleagues would understand, you know, can be difficult.”* (Participant 8, locally born)

Another uncertainty signal observed was the *behavior of SIEs*, which ten supervisors noted. This behavior often distinguishes SIEs from local employees, particularly when multiple individuals from the same nationality are present.:

*“In some cultures, I can see that cult mentality. They group, they keep secrets and information, and that is definitely there.”* (Participant 6, foreign-born)

Another signal noted by both foreign-born and locally born supervisors (11 participants) was that, generally speaking, most SIEs exhibit *strong work ethics* with only a few exceptions:

*“In general, immigrants have a stronger work ethic, maybe because of the countries they come from. <…> Based on my experience, immigrants I have hired and supervised have had a strong work ethic.”* (Participant 2, foreign-born)

This study reveals that supervisors with international experience, especially foreign-born supervisors, are more likely to recognize uncertainty signals from incoming SIEs. Locally born supervisors identified fewer uncertainty signals than their foreign-born counterparts, suggesting that foreign-born supervisors were more attuned to the challenges of SIEs.

Table 2 depicts how direct supervisors interpret uncertainty signals coming from SIEs. Five main themes were identified: comparing one’s own adjustment experience, making assumptions, perceiving a need to build trust, appreciating the situation, and perceiving SIEs’ insecurities.

**Table 2:** The codes of the direct supervisor’s interpretation of signals.

Topics of interpretation	Foreign-born supervisors		Locally-born supervisor	
	Individuals reporting the reference	Number of references made	Individuals reporting the reference	Number of references made
**Compared to own adjustment experience**	8	18	1	2
**Making assumptions**				
Generalizing toward nationalities	8	15	1	2
Noticing biases because of different cultures	6	9	2	4
**Perceiving a need to build trust**	6	9	3	3
**Appreciating the situation**				
Appreciation from the direct supervisor	6	7	2	3
Appreciation from SIE	1	1	1	1
**Perceiving SIEs’ insecurities**	4	4	0	0

One of the most notable interpretations made by foreign-born supervisors is *comparing their own adjustment experiences* (as reported by 8 respondents). Based on that, all participants recognized the warning signals of SIEs’ need for assistance or support for their adjustment, either in the work or non-work environment:

*“I have an empathy for folks that are immigrants over here, just having gone through it myself, I think a lot of the challenges are very similar with immigrants.”* (Participant 5, foreign-born)

Another interpretation, mainly by foreign-born supervisors, was making assumptions by *generalizing individuals’ behavior toward nationalities* (9 interviewees):

*“<…> I have also been in nursing school with Nigerian nurses who were learning to be nurses. Some of them were okay, but most of them had unappealing personalities. There have been multiple instances where items were not completed for the next shift. <…> [Next time] somebody else comes in, and you can see that they are also from that region, and right away, you think, ‘OK.’ Hopefully, it will be a better day.* (Participant 13, foreign-born)

In addition, only foreign-born supervisors voiced the *insecurities* that SIEs face (4 participants):

*“Asking for stuff and asking for help are also issues. When people do not know how to ask for help or are afraid to ask for help due to a language barrier, they often feel that their skills are lacking.* (Participant 13, foreign-born)

The results highlight that many foreign-born supervisors generalize their experiences (positive or negative) with a particular individual, attributing them to their nationality and projecting that generalization toward workers from the same origin. It may stem from their own experience as foreigners, where they recognize that they are different from locals; therefore, they attribute other people’s differences to their country rather than individuality**.** Additionally, foreign-born supervisors are more likely to recognize biases that stem from different cultural backgrounds. More research is needed to understand why foreign-born supervisors are more judgmental than locally born direct supervisors and why they feel insecure in the US despite having achieved substantial careers there. The following questions arise: Do foreign-born supervisors have preconceptions that any deviation from behavioral norms must be attributed to cultural differences? Do locally born supervisors, despite having international experience, exhibit fewer cultural preconceptions and biases, or are they less forthcoming about this in interviews?

Additionally, participants highlighted distinct cultural groups, such as Asians or Europeans, and separate countries, including Eritrea or France. It is unclear whether the direct supervisors’ observations of SIEs from different nations on the same continent would differ if few were on the same team. Additionally, we have not identified a pattern indicating which group of participants generalized their SIEs by continent versus country. More research is needed to analyze the generalization factors.

Foreign-born direct supervisors provided a wider variety of interpretations than locally born direct supervisors, highlighting that direct supervisors who immigrated and had to adjust are more attuned to the SIEs’ adjustment issues than those with no firsthand experience.

**What reactions do uncertainty signals elicit in a direct supervisor, and how does the direct supervisor respond to those reactions?**

Table 3 illustrates the direct supervisors’ actions in response to interpreting the SIEs’ uncertainty signals, thereby supporting their cross-cultural adjustment. We grouped the results into three categories of support for SIEs’ adjustments: work, general, and interaction adjustments.

**Table 3:** The codes of the direct supervisor’s actions in response to uncertainty signals.

Actions in response to uncertainty signals	Foreign-born supervisor		Locally-born supervisor	
	Individuals reporting the reference	Number of references made	Individuals reporting the reference	Number of references made
**Support for work adjustment**				
Training help	5	6	2	3
Helping with work situations	4	5	2	2
Motivating, encouraging	2	3	1	1
**Support for general adjustment**				
Mentoring	3	3	2	2
Making efforts to improve SIE confidence	2	2	1	1
**Support for interaction adjustment**				
Making efforts to understand SIE better	6	9	4	5
Making efforts to improve communication	3	5	0	0
Getting to know SIE better	2	2	2	2

Supervisors’ actions to support SIEs’ work adjustment primarily manifest in *training help* (7 participants). This result is not surprising and may not be directly related to international experience, as a supervisor’s primary function at work is to train incoming employees to perform their duties effectively.

*“One of them had a lot more issues that we had to purchase a special program for her to check her spell check because she could just not do it.”* (Participant 1, foreign-born)

Only five direct supervisors noted that they advise on general adjustment. They mostly see themselves *as mentors to* their SIEs. As the supervisory function is work-related, the results were not surprising:

*“Give them advice on things, show them how things work financially in America, how to build their credit. Stuff like that. Like housing.* (Participant 11, locally born)

All direct supervisors paid close attention to facilitating interaction adjustments. However, only foreign-born supervisors have noted *efforts to improve communication* (3).

*“I can adjust my own personal communication style based on where people are coming from. Speak more directly to some people. That is one way. So, just adjusting the communication style is one way.”* (Participant 15, foreign-born)

All participants, foreign-born and locally born, were more actively listing their actions to support SIEs in work and interaction adjustments rather than general adjustments. All direct supervisors understand the responsibility of training and integrating incoming SIEs into the team, ensuring that work and interactions within the international team result in the expected performance. However, only foreign-born supervisors adjusted their communication style to achieve better communication with SIEs.

The expectation was that foreign-born supervisors would have provided more guidance to their SIEs to support their general adjustment, given the similarity of their experiences in foreign countries. That was not observed in this research: supervisors from both groups mentioned very few examples of their actions to support SIEs’ general adjustment. One reason might be that the SIE or supervisor is uncomfortable discussing personal matters in the work environment, especially with someone in a higher-level position.

**How does acting on these reactions reflect on SIE work and non-work adjustment from the perspective of the direct supervisor?**

Table 4 presents the views of direct supervisors regarding their contributions to SIE’s adjustment. The results are grouped into two dimensions: work and non-work environment.

**Table 4:** The codes of the direct supervisor’s presumed effect on SIE adjustment.

Direct supervisors’ effect on SIEs’ adjustment	Foreign-born supervisor		Locally-born supervisor	
	Individuals reporting the reference	Number of references made	Individuals reporting the reference	Number of references made
Work environment	13	17	4	8
Non-work environment	3	5	2	5

With regard to SIEs’ adjustment in the work environment, seventeen direct supervisors reported that their international experience enhances their understanding of SIEs, improves communication, facilitates conflict resolution, and contributes to a greater sense of workplace integration for SIEs:

*“I can guide them, I guess, more effectively and provide some of the methods and solutions that I have employed before for them to test it out to see if they would work for them as well.”* (Participant 5, foreign-born)

Only five direct supervisors noted that their advice and guidance might assist SIEs in their adjustment to a *non-work environment*:

*“he wants to own his own home one day and provide for things like that. So that is how I like, give them advice on things like that, but never like how to behave like he.”* (Participant 11, locally born)

The results show that foreign-born and locally born direct supervisors implied that they might affect SIEs’ adjustment more frequently in the work environment than in non-work environments.

## Discussion

Drawing on signaling [19,25] and similarity-attraction theories [20,43] this exploratory study investigated how the international experience of foreign and locally born direct supervisors intersects with and influences the adjustment of SIEs in work and non-work environments.

The findings indicate that supervisors perceive their international experience as a powerful tool to help them better understand the adjustment challenges of incoming SIEs. All participants reported noticing uncertainty signals from SIEs due to their international experience. However, locally-born supervisors with international experience were less attuned to those signals than foreign-born supervisors. This aligns with signaling theory research, which states that not all recipients interpret signals similarly [87–89]. Therefore, locally born direct supervisors with international experience might have noticed the signals but did not recognize them as uncertainty signals. On the other hand, why is the international experience of locally born supervisors insufficient to make them equal recipients of uncertainty signals? Why are they less perceptive? The scientific literature analyzed types of international experience based on the number of experiences [90,91], length [92,93], or international education [94]. Each influences individuals’ attitudes, behaviors, values, and knowledge [95]. However, people interpret situational cues depending on several factors, including past experiences [96]. Therefore, as locally born direct supervisors lack the migration experience of foreign-born supervisors, this may explain why they are less perceptive in interpreting uncertainty signals. This is also consistent with the similarity-attraction theory [20,43]. It posits that perceived similarity fosters attraction, leading to positively biased decisions and evaluations [97]. The results highlight the distinctive knowledge of foreign-born supervisors regarding cultural and regulatory differences between their home and host countries [98]. This knowledge makes them more confident in their ability to manage incoming SIEs and support their adjustment to the workplace.

Howe-Walsh and Schyns [99] emphasize that direct supervisors play a crucial role in implementing HR policies and should be provided with various support strategies for working with SIEs. Our research suggests that the reverse may also be true: direct supervisors with international experience, especially foreign-born supervisors, can be valuable resources for HR professionals in developing strategies to support and facilitate the adjustment of incoming SIEs in local organizations.

Foreign and locally born direct supervisors were similarly engaged in actions that supported SIEs’ work and interaction adjustments. However, they were much less engaged in supporting SIEs’ general adjustment. In addition, the results indicate that direct supervisors consider themselves more effective in facilitating SIEs’ adjustment in the work environment than in the non-work environment. Existing research provides several explanations for this phenomenon. Haslberger et al. [100] observed that direct supervisors typically refrain from involvement in subordinates’ personal matters, as non-work adjustment is perceived to fall outside their sphere of influence, control, or responsibility. Since SIEs join organisations to fulfil specific professional roles, supervisors tend to focus on their adjustment within the workplace, ensuring that they can effectively perform their tasks and promptly contribute to overall organisational performance [101]. As argued by Lazarova and Cerdin [102], although cultural integration and social adjustment are important for long-term retention, most organisations prioritise short-term performance objectives. Consequently, direct supervisors are under pressure to ensure incoming SIEs concentrate on work-related aspects, leaving broader integration and adjustment efforts to the individuals themselves. Furthermore, Tharenou [98] noted that SIEs are often perceived as more self-sufficient and independent than corporate expatriates, given that they have voluntarily relocated. As a result, supervisors may not recognise the extent to which SIEs require support in the non-work environment. More research would be beneficial, as foreign-born supervisors often have their own experiences with general adjustment and can guide new SIEs. On the other hand, locally born supervisors living in the country have local experience and may present more options for SIEs to choose from during their general adjustment.

The results suggest that foreign-born supervisors are more likely to exhibit stereotyping than locally-born supervisors. It is unclear why stereotyping or attributing biases appear prevalent among foreign-born supervisors in this research. As foreign-born supervisors are from different cultural groups than locally born supervisors and are considered a minority, they may initially view other SIEs as representatives of another cultural group, unconsciously differentiating themselves from them while generalizing them as outsiders. Berry [103] suggested that those who integrate deeply into a host country’s society and culture might distance themselves from “outsiders” to avoid being associated with them. This also aligns with social identity theory [104], which suggests that individuals tend to view themselves and others in different categories, leading to ingroup favoritism and outgroup differentiation. Therefore, if supervisors consider themselves assimilated host-country professionals, they might stereotype the incoming SIEs as ‘outsiders’ and prefer not to assist them with their adjustment. On the other hand, if supervisors identify themselves with expatriates, they, based on their own experience, may assume that incoming SIEs will struggle with adjustment and need assistance. Moreover, if supervisors had difficulties with their adjustment in the past, some may experience a contrast effect [105], expecting incoming SIEs to endure the same struggles without offering them much assistance. Stereotyped behavior might also be a product of confirmation bias [106], suggesting that previous negative experiences with supervising SIEs might have led some direct supervisors to overgeneralize the challenges they faced, assuming the incoming SIEs would fail to integrate into the new organizational culture, struggle with workplace norms, or lack the commitment to fully adjust. Further research may be beneficial to clarify this issue.

### Limitations and suggestions for future research

This study presents the views of direct supervisors with diverse international experiences and their perceptions of themselves as better equipped to manage and guide SIEs on their teams within the US context. However, this study has several limitations. First, most participants worked in the San Francisco Bay Area, considered the world’s sixth most culturally diverse population [107]. Therefore, research with samples from other areas of the US and other countries may reveal different perspectives on the importance of direct supervisors’ international experience. Moreover, comparative studies involving participants from different regions or across multiple regions could help to assess cultural differences in SIE-supervisor-SIE-employee dynamics and provide a more comprehensive understanding of the impact of direct supervisors’ international experience on the adjustment of SIEs.

Secondly, as the Bay Area is a metropolitan region, studies involving participants from rural areas may produce different findings regarding stereotyping and the distinct characteristics of work and non-work environments. Moreover, comparative studies examining the rural vs urban adjustment contexts for SIEs might lead to additional insights in the field.

Thirdly, participants emphasize separate cultural groups, such as Asians or Europeans, and separate countries, like Eritrea or France; therefore, research concentrating on SIEs from a specific cultural group or country might yield notable results.

Fourth, all study participants have some international experience; however, many direct supervisors in the local workforce generally have no international experience. Comparative research with supervisors without international experience to serve as a control group may reveal the similarities and differences in perceptions, yielding meaningful and valuable insights.

Fifth, this study presents a one-sided perspective, focusing solely on the direct supervisor. Dyadic research incorporating both perspectives of self-initiated expatriates (SIEs) and of their direct supervisor would offer a more comprehensive understanding of their adjustment in both work and non-work environments. Additionally, a multi-perspective approach could reveal not only shared perceptions of the impact of supervisors’ international experience but also potential differences in how each group interprets and understands these effects.

Finally, this study did not distinguish participants based on occupation or industry. Future research focusing on specific occupations or industries may provide alternative perspectives and reveal variations in the applicability of the findings. A comparative analysis of direct supervisors’ international experience across different sectors (e.g., manufacturing versus services) could offer deeper insights into the specific challenges and dynamics of SIEs’ adjustment.

### Theoretical contributions

This study presents an original analysis of the international experiences of foreign and locally born direct supervisors. To the best of the authors’ knowledge, this is the first empirical study to investigate how the international experience of a direct supervisor affects the adjustment of incoming SIEs. The results revealed direct supervisors’ inclinations towards assisting SIEs in adjusting to the workplace, thereby uncovering a research gap that requires further attention from international human resource management researchers. This study paves the way for future research to delve deeper into this area, notably by addressing a few unanswered questions: (i) What are the differences between direct supervisors’ effect on SIEs’ work and non-work adjustment in metropolitan and rural areas of the US, different US states, or different countries? (ii) What are the similarities and differences between direct supervisors’ perceptions, interpretations, and actions upon uncertainty signals when all SIEs are of the same nationality as their direct supervisor, compared to when all SIEs are of the same nationality but the direct supervisor is of a different nationality? (iii) How would direct supervisors without international experience detect, interpret, and respond to SIEs’ uncertainty signals compared to direct supervisors with international experience? (iv) What are the similarities and differences in the effect of direct supervisors on SIE adjustment when the direct supervisor is from upper management versus a first-line supervisor?

Secondly, the results underscore the importance of uncertainty signals detected by direct supervisors, manifested in SIEs’ behavior, appearance, and actions in the new environment. Often unintentional and not directed to the supervisor, these signals can provide valuable insights into the SIEs’ adjustment process. The study aligns with signaling theory [19,25], indicating that the interpretation of the signals depends on the receiver. Foreign-born supervisors were more likely to notice uncertain signals and provide more interpretations of various signals than locally born supervisors, even though they also had some international experience. This highlights the unique application of signaling theory [19,25] in specific circumstances, which is more clearly understood in conjunction with similarity-attraction theory [20,43]. Drawing on their unique international experience, foreign-born supervisors often find SIEs more relatable and attractive. This shared background fosters greater support for SIEs, making them appear more approachable.

### Managerial contributions and recommendations for practice

This study advances our understanding of self-initiated expatriates’ (SIEs) interactions with local supervisors and their adjustment. It offers valuable insights for US employers, HRM professionals, foreign and locally born supervisors, and SIEs themselves.

For US employers, the findings underscore an undeniable trend: the increasing diversity of the workforce, which now includes a growing number of SIEs in supervisory and managerial roles. This emerging talent pool contributes significantly to the local economy, yet it also necessitates changes in recruitment, integration, and ongoing management practices. While local employees are expected to be familiar with local values and workplace regulations, such assumptions should not extend to foreign-born supervisors and employees. This calls for an initial effort in providing information and fostering acculturation, without undermining foreign workers’ valuable skills, such as knowledge of different cultures (and languages), a strong sense of agency, and work ethics. Building a cohesive yet diverse workforce is both a requirement and a worthwhile investment for organisations.

The findings also highlight the need for a more proactive and strategic role for HRM professionals. Locally managing an internationally diverse workforce requires additional HRM tools, including regular communication, cross-cultural support, training, and culturally diverse mentoring. HR professionals should first acknowledge the unique challenges faced by incoming SIEs and provide them with practical examples of different approaches to managing changing dynamics within teams.

For local supervisors, the findings serve as a cautionary note regarding the potential impact of stereotypes about foreign nationals on their attitudes and behaviours towards foreign-born workers. Despite efforts to adopt a personalised approach, such supervisors may inadvertently overlook foreign employees’ challenges, including their unique strengths. This could increase team friction, ultimately affecting individual and team performance.

For foreign-born supervisors, the findings reinforce the importance of recognising signs of misadjustment and well-being issues among other SIEs. Supervisors are encouraged to take proactive steps rather than neglect or minimize these signs, leveraging their international experience to support SIEs and foster a more inclusive and high-performing work environment.

Lastly, for SIEs working in the US, the findings present both disheartening and optimistic perspectives. On the one hand, the results confirm what many SIEs already feel: the lack of support for their misadjustment and ill-being, particularly if they do not communicate these challenges clearly. On the positive side, the study also highlights that those who have previously experienced similar challenges are the most likely to empathise with SIEs’ difficulties and provide meaningful support. These individuals, even in supervisory or managerial roles, can offer valuable assistance. This agency, in turn, leads to greater inclusivity, support, and smoother work adjustment for SIEs.

## Conclusion

The primary purpose of this exploratory study was to understand how the international experience of local and foreign-born supervisors intersects with SIEs’ adjustment in both work and non-work environments. Drawing on signaling [19,25] and similarity-attraction theories [20,43], a qualitative study was conducted with 20 direct supervisors with varied international experiences and at least one SIE. The results show that direct supervisors consider their international experience an additional tool for managing their international team and assisting SIEs under their command in adjusting to the work environment. The findings further revealed that foreign-born supervisors detect more uncertainty signals from SIEs and provide broader interpretations based on their international experience and their similarity with incoming SIEs. Participants noted that they are more open-minded, observant, and considerate towards SIEs because they are more knowledgeable about different cultures and personal migration experiences than their work colleagues; therefore, they are better equipped to assist incoming SIEs during their adjustment challenges.

As migration increases, more SIEs are entering local organizations. The study indicates that when these employees have foreign-born supervisors with international experience, their adjustment process is notably smoother, enabling them to integrate and contribute to the organization more effectively and in a shorter timeframe.

## Supporting information

S1 Appendix The interview protocol. (DOCX)

S2 Appendix General information about participants in the study. (DOCX)

## References

[1] Cerdin J-L, Selmer J. Who is a self-initiated expatriate? Towards conceptual clarity of a common notion. The International Journal of Human Resource Management. 2014;25(9):1281–301. doi: 10.1080/09585192.2013.863793

[2] Andreason AW. Expatriate adjustment to foreign assignments. International Journal of Commerce and Management. 2003;13(1):42–60. doi: 10.1108/eb047459

[3] Shaffer M, Harrison D, Gilley K. Dimensions, determinants, and differences in the expatriate adjustment process. Journal of International Business Studies. 1999;30(3):557–81.

[4] Toh SM, Denisi AS. Host country nationals as socializing agents: A social identity approach. Journal of Organizational Behavior. 2007;28(3):281–301.

[5] Hussain T, Zhang Y. The influences of cross-cultural adjustment and motivation on self-initiated expatriates’ innovative work behavior. Personnel Review. 2021.

[6] Kawai N, Mohr A. The contingent effects of role ambiguity and role novelty on expatriates’ work-related outcomes. British Journal of Management. 2015;26(2):163–81.

[7] Stroppa C, Spieß E. International assignments: The role of social support and personal initiative. International Journal of Intercultural Relations. 2011;35(2):234–45.

[8] Salomaa R. Expatriate coaching: factors impacting coaching success. Journal of Global Mobility. 2015;3(3):216–43. doi: 10.1108/jgm-10-2014-0050

[9] Wang X, Nayir DZ. How and when is social networking important? Comparing European expatriate adjustment in China and Turkey. Journal of International Management. 2006;12(4):449–72.

[10] Froese F, Kim H, Engle R. Expatriate management in South Korea: The role of host country context. Asia Pacific Journal of Management. 2020;37(1):185–206.

[11] Kang H, Shen J, Xu G. International training and management development policies and practices of South Korean MNEs in China. Thunderbird International Business Review. 2015;57(3):229–40.

[12] Sokro E, Pillay S, Bednall T. The effects of perceived organisational support on expatriate adjustment, assignment completion and job satisfaction. International Journal of Cross Cultural Management. 2021;21(3):452–73.

[13] Sokro E, Moeti-Lysson J. The role of host country nationals’ support in expatriate adjustment and assignment success : a case of Ghana. AJBER. 2018;13(3):95–113. doi: 10.31920/1750-4562/2018/v13n3a5

[14] Okpara J, Kabongo J. The effect of cross-cultural training on expatriates’ adjustment: Evidence from an emerging African economy. Journal of Management Development. 2017;36(9):1114–24.

[15] Kaur A, Maheshwari S, Varma A. Sailing through the international assignment: exploring the role of perceived credibility in expatriate adjustment and socialization process in the host country. Journal of Global Mobility: The Home of Expatriate Management Research. 2024;12(3):502–19.

[16] Froese FJ, Peltokorpi V. Organizational expatriates and self-initiated expatriates: differences in cross-cultural adjustment and job satisfaction. The International Journal of Human Resource Management. 2013;24(10):1953–67. doi: 10.1080/09585192.2012.725078

[17] Samarsky E. Exploring the impact of national context on adjustment of self-initiated expatriates: the case of German professionals in Britain. Career Development International. 2023;28(4):458–72.

[18] Isakovic A, Forseth Whitman M. Self-initiated expatriate adjustment in the United Arab Emirates: a study of academics. Journal of Global Mobility. 2013;1(2):161–86.

[19] Spence M. Job Market Signaling. The Quarterly Journal of Economics. 1973;87(3):355. doi: 10.2307/1882010

[20] Byrne D. An Overview (and Underview) of Research and Theory within the Attraction Paradigm. Journal of Social and Personal Relationships. 1997;14(3):417–31. doi: 10.1177/0265407597143008

[21] Vromans RD, van de Ven CCM, Willems SJW, Krahmer EJ, Swerts MGJ. It is, uh, very likely? The impact of prosodic uncertainty cues on the perception and interpretation of spoken verbal probability phrases. Risk Anal. 2024;44(10):2496–515. doi: 10.1111/risa.14319 38742599

[22] Betz S, Zarrieß S, Székely É, Wagner P. The greennn tree - lengthening position influences uncertainty perception. 2019. 3990–4.

[23] Krahmer EJ, Swerts MGJ. Signaling and detecting uncertainty in audiovisual speech by children and adults. In: Proceedings of the 8th International Conference on Spoken Language Processing (ICSLP). ISCA; 2004 [cited 2025 May 24]. 4. https://research.tilburguniversity.edu/files/675957/signaling.pdf

[24] Hunyadi L. Uncertainty in Conversation: Its Formal Cues Across Modalities and Time. In: Hunyadi L, SzekrényeS I. The Temporal Structure of Multimodal Communication. Cham: Springer International Publishing; 2020 [cited 2025 May 24]. 113–35. http://link.springer.com/10.1007/978-3-030-22895-8_6

[25] Connelly BL, Certo ST, Ireland RD, Reutzel CR. Signaling Theory: A Review and Assessment. Journal of Management. 2011;37(1):39–67. doi: 10.1177/0149206310388419

[26] Davila A, Foster G, Gupta M. Venture capital financing and the growth of startup firms. Journal of Business Venturing. 2003;18(6):689–708.

[27] Johnstone R, Grafen A. Dishonesty and the handicap principle. Animal Behaviour. 1993;46(4).

[28] Ndofor HA, Levitas E. Signaling the Strategic Value of Knowledge. Journal of Management. 2004;30(5):685–702.

[29] Cohen BD, Dean TJ. Information asymmetry and investor valuation of IPOs: top management team legitimacy as a capital market signal. Strategic Management Journal. 2005;26(7):683–90.

[30] Gulati R, Higgins MC. Which ties matter when? the contingent effects of interorganizational partnerships on IPO success. Strategic Management Journal. 2003;24(2):127–44. doi: 10.1002/smj.287

[31] Ilmola L, Kuusi O. Filters of weak signals hinder foresight: Monitoring weak signals efficiently in corporate decision-making. Futures. 2006;38(8):908–24.

[32] Bangerter A, Roulin N, König CJ. Personnel selection as a signaling game. J Appl Psychol. 2012;97(4):719–38. doi: 10.1037/a0026078 22040263

[33] Muduli A, Trivedi JJ. Recruitment methods, recruitment outcomes and information credibility and sufficiency. BIJ. 2020;27(4):1615–31. doi: 10.1108/bij-07-2019-0312

[34] Vogel D, Döring M, Sievert M. Motivational signals in public sector job advertisements and how they relate to attracting and hiring candidates. Public Management Review. 2023;26(10):2868–900. doi: 10.1080/14719037.2023.2291068

[35] Broschak JP, Block ES, Koppman S, Adjerid I. Will We Ever Meet Again? The Relationship between Inter‐Firm Managerial Migration and the Circulation of Client Ties. J Management Studies. 2020;57(6):1106–42. doi: 10.1111/joms.12522

[36] Guest DE, Sanders K, Rodrigues R, Oliveira T. Signalling theory as a framework for analysing human resource management processes and integrating human resource attribution theories: A conceptual analysis and empirical exploration. Human Res Mgmt Journal. 2021;31(3):796–818. doi: 10.1111/1748-8583.12326

[37] Yasar B, Martin T, Kiessling T. An empirical test of signalling theory. MRR. 2020;43(11):1309–35. doi: 10.1108/mrr-08-2019-0338

[38] Cañibano A, Avgoustaki A. To telework or not to telework: does the macro context matter? A signalling theory analysis of employee interpretations of telework in times of turbulence. Human Resource Management Journal. 2022.

[39] Teng-Calleja M, Presbitero A, de Guzman MM. Organizational direction, expectations, and employees’ intention for Green HRM practices in the Philippines: a signaling theory perspective. Asian Bus Manage. 2023;22(4):1301–27. doi: 10.1057/s41291-022-00206-1

[40] Venkataramani V, Bartol KM, Zheng X, Lu S, Liu X. Not very competent but connected: Leaders’ use of employee social networks as prisms to make delegation decisions. J Appl Psychol. 2022;107(3):458–80. doi: 10.1037/apl0000902 34096748

[41] Mahnke V, Ambos B, Nell PC, Hobdari B. How do regional headquarters influence corporate decisions in networked MNCs?. Journal of International Management. 2012;18(3):293–301.

[42] Taj SA. Application of signaling theory in management research: Addressing major gaps in theory. European Management Journal. 2016;34(4):338–48.

[43] Byrne DE. The attraction paradigm. Academic Press; 1971. 462.

[44] Duck SW, Craig G. Personality similarity and the development of friendship: A longitudinal study. Br J Soc Clin Psychol. 1978;17(3):237–42. doi: 10.1111/j.2044-8260.1978.tb00272.x

[45] Ruijten PAM. The similarity-attraction paradigm in persuasive technology: effects of system and user personality on evaluations and persuasiveness of an interactive system. Behaviour & Information Technology. 2021;40(8):734–46. doi: 10.1080/0144929x.2020.1723701

[46] Takeuchi R, Chen J. The impact of international experiences for expatriates’ cross-cultural adjustment. Organizational Psychology Review. 2013;3(3):248–90. doi: 10.1177/2041386613492167

[47] Takeuchi R, Wang M, Marinova S. Antecedents and consequences of psychological workplace strain during expatriation: A cross-sectional and longitudinal investigation. Personnel Psychology. 2005;58(4):925–48.

[48] Dimitrova M, Chia SI, Shaffer MA, Tay-Lee C. Forgotten travelers: Adjustment and career implications of international business travel for expatriates. Journal of International Management. 2020;26(1).

[49] Grill S, Rosenbaum-Feldbrügge M, Fliege H, Rüger H. Expatriate adjustment over time among foreign service employees: the role of cross-cultural experience. JGM. 2021;9(3):338–60. doi: 10.1108/jgm-01-2021-0009

[50] Fenner CR Jr, Selmer J. Public sector expatriate managers: psychological adjustment, personal characteristics and job factors1. The International Journal of Human Resource Management. 2008;19(7):1237–52. doi: 10.1080/09585190802110026

[51] Buengeler C, Leroy H, De Stobbeleir K. How leaders shape the impact of HR’s diversity practices on employee inclusion. Human Resource Management Review. 2018;28(3):289–303.

[52] Den Hartog DN, Boon C, Verburg RM, Croon MA. HRM, Communication, Satisfaction, and Perceived Performance. Journal of Management. 2013;39(6):1637–65. doi: 10.1177/0149206312440118

[53] Gilbert C, De Winne S, Sels L. Strong HRM processes and line managers’ effective HRM implementation: a balanced view. Human Res Mgmt Journal. 2015;25(4):600–16. doi: 10.1111/1748-8583.12088

[54] Weick K, Weick E. The social psychology of organizing. M@n@gement. 2015;18:189.

[55] Ashforth BE, Saks AM, Lee RT. Human Relations. 1998;51(7):897–926. doi: 10.1023/a:1016999527596

[56] Bauer TN, Erdogan B, Caughlin D, Ellis AM, Kurkoski J. Jump-Starting the Socialization Experience: The Longitudinal Role of Day 1 Newcomer Resources on Adjustment. Journal of Management. 2021;47(8):2226–61. doi: 10.1177/0149206320962835

[57] Rubenstein AL, Kammeyer-Mueller JD, Thundiyil TG. The comparative effects of supervisor helping motives on newcomer adjustment and socialization outcomes. J Appl Psychol. 2020;105(12):1466–89. doi: 10.1037/apl0000492 32162951

[58] Chen TJ, Wu CM. Can newcomers perform better at hotels? Examining the roles of transformational leadership, supervisor-triggered positive affect, and perceived supervisor support. Tourism Management Perspectives. 2020;33:100587.

[59] Ellis AM, Nifadkar SS, Bauer TN, Erdogan B. Newcomer adjustment: Examining the role of managers’ perception of newcomer proactive behavior during organizational socialization. J Appl Psychol. 2017;102(6):993–1001. doi: 10.1037/apl0000201 28277724

[60] Jokisaari M, Vuori J. Leaders’ resources and newcomer socialization: the importance of delegation. JMP. 2018;33(2):161–75. doi: 10.1108/jmp-09-2016-0274

[61] Nelson DL, Quick JC. Social support and newcomer adjustment in organizations: Attachment theory at work?. J Organ Behavior. 1991;12(6):543–54. doi: 10.1002/job.4030120607

[62] Zhang Y, Liao J, Yan Y, Guo Y. Newcomers’ Future Work Selves, Perceived Supervisor Support, and Proactive Socialization in Chinese Organizations. Social Behavior and Personality: an international journal. 2014;42(9):1457–72.

[63] Korte R, Brunhaver S, Sheppard S. (Mis)Interpretations of Organizational Socialization: The Expectations and Experiences of Newcomers and Managers. Human Resource Development Quarterly. 2015;26(2):185–208. doi: 10.1002/hrdq.21206

[64] Dufour L, Maoret M, Montani F. Coupling High Self‐Perceived Creativity and Successful Newcomer Adjustment in Organizations: The Role of Supervisor Trust and Support for Authentic Self‐Expression. J Management Studies. 2020;57(8):1531–55. doi: 10.1111/joms.12547

[65] Boyd NG, Taylor RR. A developmental approach to the examination of friendship in leader-follower relationships. 1998.

[66] Andresen M, Bergdolt F, Margenfeld J. What distinguishes self-initiated expatriates from assigned expatriates and migrants?: A literature-based definition and differentiation of terms. Self-Initiated Expatriation: Individual, Organizational, and National Perspectives. 2012. 11–41.

[67] Przytuła S. Migrants, assigned expatriates (AE) and self-initiated expatriates (SIE) - differentiation of terms and literature-based research review. Journal of Intercultural Management. 2015;7(2):89–111.

[68] Suutari V, Brewster C, Mäkelä L, Dickmann M, Tornikoski C. The Effect of International Work Experience on the Career Success of Expatriates: A Comparison of Assigned and Self‐Initiated Expatriates. Human Resource Management. 2018;57(1):37–54. doi: 10.1002/hrm.21827

[69] van Bakel M. It takes two to tango: a review of the empirical research on expatriate-local interactions. The International Journal of Human Resource Management. 2019;30(21):2993–3025. doi: 10.1080/09585192.2018.1449763

[70] Jonasson C, Lauring J, Guttormsen D. Inclusive management in international organizations: How does it affect local and expatriate academics?. Personnel Review. 2018;47(2):458–73.

[71] Syed J, Hazboun N, Murray P. What locals want: Jordanian employees’ views on expatriate managers. International Journal of Human Resource Management. 2014;25(2):212–33.

[72] Tsai CJ, Carr C, Qiao K, Supprakit S. Modes of cross-cultural leadership adjustment: adapting leadership to meet local conditions and/or changing followers to match personal requirements? International Journal of Human Resource Management. 2019;30(9):1477–504.

[73] Patton MQ. Qualitative Research & Evaluation Methods: Integrating Theory and Practice. SAGE Publications; 2014. 833.

[74] Bluhm DJ, Harman W, Lee TW, Mitchell TR. Qualitative Research in Management: A Decade of Progress. J Management Studies. 2011;48(8):1866–91. doi: 10.1111/j.1467-6486.2010.00972.x

[75] Lyu Y. Shifting research paradigms in educational research: From positivism to interpretivism. Science, Technology and Social Development Proceedings Series. 2024;2:11–4.

[76] Van der Walt JL. Interpretivism-constructivism as a research method in the humanities and social sciences–more to it than meets the eye. International Journal of Philosophy and Theology. 2020;8(1):59–68.

[77] Saunders M, Lewis P, Thornhill A. Research Methods for Business Students. Harlow: Pearson; 2019.

[78] Hennink MM, Kaiser BN, Marconi VC. Code Saturation Versus Meaning Saturation: How Many Interviews Are Enough?. Qual Health Res. 2017;27(4):591–608. doi: 10.1177/1049732316665344 27670770 PMC9359070

[79] Hennink M, Kaiser BN. Sample sizes for saturation in qualitative research: A systematic review of empirical tests. Soc Sci Med. 2022;292:114523. doi: 10.1016/j.socscimed.2021.114523 34785096

[80] Malterud K, Siersma VD, Guassora AD. Sample Size in Qualitative Interview Studies: Guided by Information Power. Qual Health Res. 2016;26(13):1753–60. doi: 10.1177/1049732315617444 26613970

[81] Wutich A, Beresford M, Bernard HR. Sample sizes for 10 types of qualitative data analysis: An integrative review, empirical guidance, and next steps. International Journal of Qualitative Methods. 2024;23.

[82] Burla L, Knierim B, Barth J, Liewald K, Duetz M, Abel T. From text to codings: intercoder reliability assessment in qualitative content analysis. Nursing Research. 2008;57(2):113–7. 18347483 10.1097/01.NNR.0000313482.33917.7d

[83] Ortloff A-M, Fassl M, Ponticello A, Martius F, Mertens A, Krombholz K, et al. Different Researchers, Different Results? Analyzing the Influence of Researcher Experience and Data Type During Qualitative Analysis of an Interview and Survey Study on Security Advice. In: Proceedings of the 2023 CHI Conference on Human Factors in Computing Systems, 2023. 1–21. doi: 10.1145/3544548.3580766

[84] Frankfort-Nachmias C, Nachmias D. Study Guide for Research Methods in the Social Sciences. Macmillan; 2007. 292.

[85] Black JS, Mendenhall M, Oddou G. Toward a comprehensive model of international adjustment: An integration of multiple theoretical perspectives. Academy of Management Review. 1991;16:291–317.

[86] Haslberger A, Brewster C, Hippler T. Managing performance abroad: A new model for understanding expatriate adjustment. Routledge; 2014. 209.

[87] Highhouse S, Thornbury EE, Little IS. Social-identity functions of attraction to organizations. Organizational Behavior and Human Decision Processes. 2007;103(1):134–46.

[88] Suazo MM, Martínez PG, Sandoval R. Creating psychological and legal contracts through human resource practices: A signaling theory perspective. Human Resource Management Review. 2009;19(2):154–66.

[89] Xu M, Qin X, Dust SB, DiRenzo MS. Supervisor-subordinate proactive personality congruence and psychological safety: A signaling theory approach to employee voice behavior. The Leadership Quarterly. 2019;30(4):440–53.

[90] Daily CM, Certo ST, Dalton DR. International experience in the executive suite: the path to prosperity?. Strategic Management Journal. 2000;21(4):515–23.

[91] Shaffer MA, Harrison DA, Gilley KM. Dimensions, Determinants, and Differences in the Expatriate Adjustment Process. J Int Bus Stud. 1999;30(3):557–81. doi: 10.1057/palgrave.jibs.8490083

[92] Gregersen HB, Black JS. Antecedents to Commitment to a Parent Company and a Foreign Operation. Academy of Management Journal. 1992;35(1):65–90.

[93] Parker B, McEvoy GM. Initial examination of a model of intercultural adjustment. International Journal of Intercultural Relations. 1993;17(3):355–79.

[94] Tihanyi L, Ellstrand AE, Daily CM, Dalton DR. Composition of the top management team and firm international diversification. Journal of Management. 2000;26(6):1157–77.

[95] Takeuchi R, Tesluk P, Yun S, Lepak D. An integrative view of international experience. Academy of Management Journal. 2005;48(1):85–100.

[96] Tekoppele JL, De Hooge IE, van Trijp HCM. We’ve got a situation here! – How situation-perception dimensions and appraisal dimensions of emotion overlap. Personality and Individual Differences. 2023;200:111878.

[97] Turban DB, Jones AP. Supervisor-subordinate similarity: types, effects, and mechanisms. J Appl Psychol. 1988;73(2):228–34. doi: 10.1037/0021-9010.73.2.228 3384773

[98] Tharenou P. Self-initiated expatriates: an alternative to company-assigned expatriates?. J of Global Mobility. 2013;1(3):336–56. doi: 10.1108/jgm-02-2013-0008

[99] Howe-Walsh L, Schyns B. Self-initiated expatriation: implications for HRM. The International Journal of Human Resource Management. 2010;21(2):260–73. doi: 10.1080/09585190903509571

[100] Haslberger A, Brewster C, Hippler T. Dynamics of adjustment. 2014. 105.

[101] Peltokorpi V, Froese F. Organizational expatriates and self-initiated expatriates: who adjusts better to work and life in Japan?. International Journal of Human Resource Management. 2009;20(5):1096–112.

[102] Lazarova MB, Cerdin J-L. Revisiting repatriation concerns: organizational support versus career and contextual influences. J Int Bus Stud. 2007;38(3):404–29. doi: 10.1057/palgrave.jibs.8400273

[103] Berry JW. Constructing and Expanding a Framework: Opportunities for Developing Acculturation Research. Applied Psychology. 1997;46(1):62–8. doi: 10.1111/j.1464-0597.1997.tb01095.x

[104] Tajfel H, Turner JC, Austin WG, Worchel S. An integrative theory of intergroup conflict. Organizational identity: A reader. 1979. 56.

[105] Wilder DA, Thompson JE. Assimilation and contrast effects in the judgments of groups. Journal of Personality and Social Psychology. 1988;54(1):62–73. 10.1037//0022-3514.73.2.254 9248048

[106] Nickerson RS. Confirmation Bias: A Ubiquitous Phenomenon in Many Guises. Review of General Psychology. 1998;2(2):175–220. doi: 10.1037/1089-2680.2.2.175

[107] World Population by Country (Live). 2024 [cited 2024 May 5]. https://worldpopulationreview.com/

