# Supplementary material for: International experience of a direct supervisor–does it matter for self-initiated expatriates’ adjustment?
Source: PLoS One. 2025 Jun 23;20(6):e0326848. doi: 10.1371/journal.pone.0326848 (PMC12184935; doi:10.1371/journal.pone.0326848)
Supplement: S1 Appendix — (DOCX) [file pone.0326848.s001.docx]

| **Research Question** | **Subsidiary Research Question** | **Topic/Themes** | **Questions to supervisors** |
| --- | --- | --- | --- |
| **Respondents' Demographics \| Present Situation** | What kind of international experience do direct supervisors have? | Birth Country | Have you been born in this country? If not, how long have you been in this country? Do you have any other nationality? |
|  |  | Work experience, studies abroad, international travel, family connections, ancestry | Do you have any international experience? What kind? When? |
|  | What is the international composition of the work team? | Team composition | Do you have subordinates from different countries? Can you list all of their nationalities? |
|  | How can supervisors notice the signals related to international employee adjustment in the organization? | Initial difficulties | What are the main difficulties your international employees face when joining the company/your team? Why? Could you give examples? |
|  |  | Differences between local and international employees | Have you noticed any differences between local and international employees? What kind of differences? Why? Can you give examples? |
|  |  |  | Have you noticed any differences in your approach to local and international employees? Which ones? Why? Can you give examples? |
| **How does the international experience of a direct supervisor affect SIEs’ adjustment in work and non-work environments?** | How/in what ways does direct supervisors’ international experience affect SIEs' adjustment? | Work adjustment | In what ways is your international experience influential in your work environment? Could you give examples? Why is that? |
|  |  | Interaction adjustment | In reflecting on your international experience, in what ways does it affect your interactions with subordinates? All equally, or do you notice different reactions from them? Why? Could you give examples? |
|  |  |  | Do you think would it affect international employee adjustment if you had no international experience? How? Why? |
|  |  |  | You have some employees from different countries and some who have not. Reflecting on your international experience, how does it affect how you interact with international employees versus local employees? Why? Can you give a few examples? |
|  |  | General adjustment | What about general adjustment outside the organization? Do international employees come to you for advice? |
|  |  | Work and non-work environment | How have you/are you influencing your employees’ work and non-work adjustments? |
