# Supplementary material for: International experience of a direct supervisor–does it matter for self-initiated expatriates’ adjustment?
Source: PLoS One. 2025 Jun 23;20(6):e0326848. doi: 10.1371/journal.pone.0326848 (PMC12184935; doi:10.1371/journal.pone.0326848)
Supplement: S2 Appendix — (DOCX) [file pone.0326848.s002.docx]

| **Random number assigned to a participant** | **Gender** | **Nationality** | **Place of residence/work** | **Age group** | **How many years lived in the US (only if you not born in the US)** | **How many years worked in the US** | **How many years in a managing role with international employees on the team** | **What is the highest education level attained** |
| --- | --- | --- | --- | --- | --- | --- | --- | --- |
| 1 | F | Lithuanian | SF Bay Area | Over 50 | Over 30 | 20 | 5-9 | Graduate |
| 2 | M | Filipino | SF Bay Area | Over 50 | Over 30 | 30 | over 10 | Undergraduate |
| 3 | M | American | SF Bay Area | 40-49 | Born | 25 | 5-9 | Graduate |
| 4 | F | Salvadorian | SF Bay Area | 40-49 | Over 30 | 25 | over 10 | Undergraduate |
| 5 | F | Singaporean | SF Bay Area | 30-39 | 10-19 | 10 | over 10 | Graduate |
| 6 | M | Iranian | SF Bay Area | Over 50 | Over 30 | 35 | over 10 | Undergraduate |
| 7 | F | Lithuanian | SF Bay Area | 30-39 | 10-19 | 14 | up to 5 | Graduate |
| 8 | M | American | SF Bay Area | 40-49 | Born | 30 | 5-9 | High School |
| 9 | F | American | SF Bay Area | Over 50 | Born | 35 | up to 5 | Graduate |
| 10 | F | Lithuanian | SF Bay Area | Over 50 | 20-29 | 23 | 5-9 | Graduate |
| 11 | M | American | Maryland | 30-39 | Born | 25 | over 10 | High School |
| 12 | M | American | SF Bay Area | 40-49 | Born | 31 | 5-9 | Undergraduate |
| 13 | M | Lithuanian | Chicago Area | 30-39 | 20-29 | 18 | up to 5 | Undergraduate |
| 14 | F | Lithuanian | Chicago Area | Over 50 | 20-29 | 25 | 5-9 | Undergraduate |
| 15 | M | Lithuanian | SF Bay Area | 30-39 | 10-19 | 14 | over 10 | Graduate |
| 16 | F | Salvadorian | SF Bay Area | 40-49 | Over 30 | 15 | over 10 | Graduate |
| 17 | F | Indian | SF Bay Area | 30-39 | 10-19 | 12 | 5-9 | Graduate |
| 18 | M | American | Denver, Colorado | 40-49 | Born | 25 | up to 5 | High School |
| 19 | F | Lithuanian | Chicago Area | 40-49 | 20-29 | 21 | 5-9 | Undergraduate |
| 20 | M | Lithuanian | SF Bay Area | 40-49 | 20-29 | 15 | over 10 | Graduate |
